# Influence of Nonenzymatic Browning Reactions on the Digestibility and Gut Microbiota Fermentation of Starch and Protein

**DOI:** 10.1111/1541-4337.70299

**Published:** 2025-09-27

**Authors:** Wensheng Ding, Yichen Bai, Devin J. Rose

**Affiliations:** ^1^ Department of Food Science & Technology University of Nebraska–Lincoln Lincoln Nebraska USA; ^2^ Nebraska Food for Health Center University of Nebraska–Lincoln Lincoln Nebraska USA; ^3^ Department of Agronomy & Horticulture University of Nebraska–Lincoln Lincoln Nebraska USA

## Abstract

Cooking has dramatic effects on the digestion and fermentation of food components. The changes that occur to starch and protein during nonenzymatic browning (NEB) have garnered attention due to health concerns. Among these changes, Maillard reaction, caramelization, and oxidation have major effects on starch and protein digestibility, as well as gut microbiota fermentation. The purpose of this review is to discuss how NEB reactions influence the digestibility of starch and protein from food materials and how this might affect gut fermentation with an emphasis on the implications for human gut health. Different reactions that happen during NEB can alter starch and protein digestibility differently. Maillard reaction products (MRPs) can decrease starch digestibility directly by reacting with starch and indirectly by inhibiting amylolytic enzymes. MRPs have a dichotomous effect on the gut microbiome, where they simultaneously increase the production of the beneficial microbial metabolite, butyrate, while also enriching for detrimental sulfate‐reducing bacteria. A greater understanding of the effects of NEB on protein and starch digestibility and gut microbiota fermentation holds promise for advancing the development of healthier cooking techniques, potentially leading to meaningful improvements in health‐promoting foods.

## 1 Introduction

Nonenzymatic browning (NEB) encompasses primarily the Maillard reaction and caramelization, which are two crucial reactions in cooking that are responsible for the characteristic aromas, colors, and flavors that develop during, for example, baking, roasting, and grilling of mainly low‐ or intermediate‐moisture foods (Dornenburg and Page 2008). During NEB, proteins and sugars can react with each other and other compounds in foods to form a variety of degradation products (Humpf and Voss 2004). These reactions can decrease the digestibility of protein and starch, thus impacting macronutrient acquisition as well as the fermentation processes of gut bacteria in the large intestine.

NEB reactions are not only crucial for the sensory appeal of food but also contribute to its nutritional value and overall culinary experience. The studies on NEB reactions can be traced back to the 1940s, when people began to understand what NEB reactions are and their specific mechanisms. Pioneering studies before the 1960s uncovered the complex chemical reactions that produce brown colors in foods during cooking (Hodge and Rist 1953; Spark 1969; Wolfrom et al. 1948). When becoming familiar with NEB reactions, researchers start to investigate the factors that influence these reactions. Burton et al. suggested that sulfites can retard the browning of carbonyl and amino systems through their combination with the carbonyl group and unsaturated centers (Burton et al. 1963). The impact of sulfites on NEB was also reported in 1974, in which the addition of sulfur dioxide decreased browning concentrations in both glucose–glycine solution and dehydrated carrot extract. Later, Garza et al. focused on the NEB reaction in heated peach puree (with emphasis on the industry side) and researched different aspects such as physical and chemical characterization and the determination of hydroxymethylfurfural and sugars (Garza et al. 1999). Similarly, another paper also focused on practical conditions, investigating NEB formation during citrus juice storage (Roig et al. 1999). As the studies on nonenzymatic reactions continued, research on the impact of nonenzymatic reactions on digestibility emerged (Chung et al. 2011, Chung et al. 2012; Jiang, Feng, et al. 2021; Seiquer et al. 2006). In recent years, studies on gut microbiome have become a new trend, and so has research on the effects of NEB on gut microbiome (Delgado‐Andrade et al. 2017; Qu et al. 2018; Seiquer et al. 2006; J. Wang et al. 2022; W. Wang et al. 2023; Yacoub et al. 2017).

The purpose of this review is to discuss how NEB reactions influence the digestibility of starch and protein from food materials and how this might affect gut fermentation with an emphasis on the implications for human health. First, the Maillard reaction, caramelization, and oxidation reactions, with an emphasis on the changes in the structure of protein and starch, will be reviewed. Next, the influence of cooking on starch digestibility, protein digestibility, and gut microbiota composition will be summarized, along with the effects of certain compounds produced during NEB on human health.

## 2 NEB Reactions

### 2.1 Maillard Reaction

**FIGURE 1:**
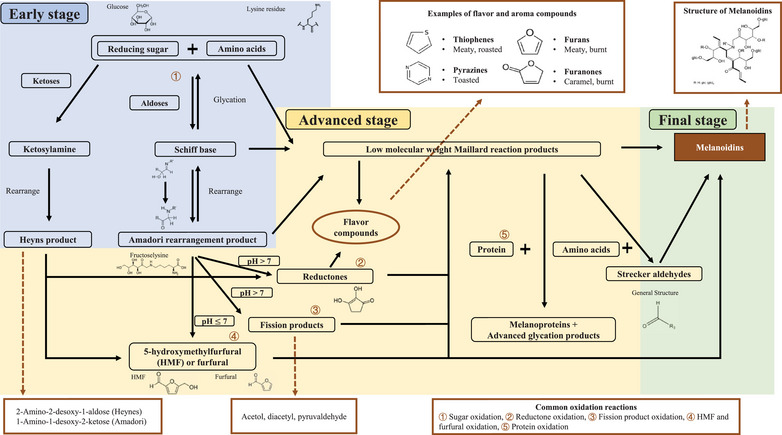
Summary of the Maillard reaction process.

The Maillard reaction is a chemical process that occurs when aldehydes, typically from reducing sugars, and amines, typically from proteins and amino acids, combine to form a variety of reaction products, including glycosylamines, Amadori compounds, and further downstream products like melanoidins (Figure 1) (Ellis 1959). This reaction is responsible for the distinct flavor and aroma found in browned food.

There are three stages in the Maillard reaction (Morales and van Boekel 1997). During the first phase, the carbonyl group of a reducing sugar reacts with the amino group of an amino compound through glycation (Schiff base formed) and rearrangement, producing Amadori products (1‐amino‐1‐deoxy‐ketose). With the ketoses, such as fructose, the ketosylamine could form and further rearrange to produce Heyns products (2‐amino‐2‐desoxialdose). This stage is reversible, and no browning is apparent (Martins et al. 2000).

In the second stage, the Amadori or Heyns products undergo further chemical transformations. These transformations involve various reactions such as sugar fragmentation and Strecker degradation. Strecker degradation is when an amino compound reacts with a dicarbonyl compound, often arising from dehydration of reducing sugar, and forms the α‐amino derivative of the dicarbonyl compound and an aldehyde derivative of the amino compound with one less carbon, which is lost as carbon dioxide (Yaylayan 2003; Yaylayan et al. 1994). The specific reactions that occur during the second stage depend on factors such as temperature, pH, and the presence of other reactants or catalysts. When the pH is equal to or less than seven, the Amadori and Heyns products mainly undergo 1,2‐enolization, forming furfural from pentoses or hydroxymethylfurfural from hexoses. When the pH is higher than seven, Amadori and Heyns products mainly undergo 2,3‐enolization, where reductones and fission products can be produced. Meanwhile, reducing sugars and amino acids, Amadori products, and Schiff base compounds can go through fragmentation, producing low‐molecular‐weight Maillard reaction products (MRPs), which are characteristic aroma compounds of the Maillard reaction. Examples of the low‐molecular‐weight aroma compounds arising from the Maillard reaction are thiophenes (meaty, roasted), furans (meaty, burnt), pyrazines (toasted), and furanone (caramel, burnt). These low‐molecular‐weight MRPs, including reductones, fission products, and furfural, can further react with protein to form melanoproteins and advanced glycation products (AGEs). They can also react with amino acids to form Strecker aldehydes (Ellis 1959).

Condensation and polymerization of low‐molecular‐weight MRPs take place in the last stage of the Maillard reaction, leading to the formation of heterocyclic nitrogenous compounds known as melanoidins, which are responsible for the visible brown colors. These MRPs can bind to proteins and are thus considered high‐molecular‐mass compounds (Lund and Ray 2017).

Numerous analytical methods have been developed to detect and quantify NEB reactions across various food matrices and stages of browning. The choice of method largely depends on the type of food, processing conditions, and the desired level of specificity. Spectrophotometric analysis at fixed wavelengths (e.g., 420 or 410 nm) remains a widely used and straightforward approach to assess browning intensity, as demonstrated in peach puree (Garza et al. 1999), dongbei suancai (C. Wang et al. 2021), and krill oil (F. S. H. Lu et al. 2014). Similarly, colorimetric measurements in the CIE Lab* color space are commonly applied to monitor visual browning in food products such as apple juice (Zhu et al. 2009), spaghetti (Doxastakis et al. 2007), and crayfish tails (Q. Jiang et al. 2023), facilitating correlations between the extent of browning and processing parameters. Fluorescence spectroscopy can also be used to distinguish browning stages, as shown in thermally treated apple juice (Zhu et al. 2009). Importantly, fluorescence measurements only detect fluorescent MRPs and provide no information on nonfluorescent MRPs or specific cross‐link structures such as protein–protein or protein–sugar adducts. These spectroscopic methods are better suited for relative comparisons rather than for quantifying individual reaction products.

For analysis of specific MRPs, more specific and structurally informative techniques have been employed. In earlier studies, MRP‐specific enzyme‐linked immunosorbent assays (ELISAs)—such as those targeting *N*‐ε‐carboxymethyllysine (CML)—were widely used. However, the poor specificity and cross‐reactivity of ELISAs have limited their reliability in complex food matrices (Poulsen et al. 2013). Nowadays, researchers use high‐performance liquid chromatography (HPLC) and liquid chromatography–mass spectrometry (LC–MS) to detect and quantify specific intermediates and products of Maillard reactions, including 5‐hydroxymethylfurfural (HMF), furosine, polyphenols, and amino nitrogen. These techniques offer high sensitivity and specificity for tracking sugar degradation and protein modification products. Gas chromatography–mass spectrometry (GC–MS) is also useful for identifying low‐molecular‐weight volatile compounds such as Strecker degradation products. For example, GC–MS was used to identify 72 volatiles in pasta subjected to different drying temperatures (Pasqualone et al. 2014).

Melanoidins, the high‐molecular‐weight brown polymers formed in the final stages of Maillard reactions, are another key indicator. Their characteristic brown color has prompted the development of colorimetric methods for rapid melanoidin quantification. A well‐known example is the extinction coefficient of 1.0 L/(mol·cm) established by Martins and Boekel for melanoidin in the glucose/glycine system, which has been widely adopted (Ding 2023; Ding et al. 2024; Martins and Boekel 2003; Silva et al. 2016). Additionally, acrylamide—another product of Maillard reactions—has drawn significant attention due to its potential health risks. Analytical protocols for acrylamide typically involve the addition of an internal standard, aqueous extraction, purification using solid‐phase extraction, and quantification via GC–MS (after derivatization) or direct LC–MS analysis (Castle and Eriksson 2005). Table 1 summarizes representative analytical methods used for NEB determination. In summary, while colorimetric and absorbance‐based methods offer simplicity and broad applicability, chromatographic, fluorescence, and volatile compound analyses provide deeper insight into the specific chemical transformations underlying NEB reactions.

**TABLE 1:** Examples of methods used for determining the NEB from foods.

Food sample	Detection methods	Equipment	Results	NEB reaction	NEB products	Advantages/disadvantages	Reference
Fresh apple juice, heated at 95°C for 30 and 60 min, stored for 6 days	Fluorescence EEM, nonenzymatic browning index (NEBI), L*a*b*, HMF	Fluorescence spectrometer, colorimeter, spectrophotometer	Fluorescence classified fresh/processed juice with >85% accuracy; NEBI, b*, and HMF all correlated >80%	Maillard reaction, ascorbic acid degradation	HMF, browning pigments	Fluorescence: sensitive and rapid, but requires calibration; Color/HMF: quantitative but less specific	Zhu et al. 2009
Peach puree, heated at 80°C–98°C (480 min)	Absorbance at 420 nm, L*a*b*, sugar and HMF content	Spectrophotometer, HPLC	b* decreased, a* increased with heat; NEB intensified with temperature; kinetic correlation with temperature	Maillard reaction, caramelization	HMF, browning pigments	Spectrophotometry: easy and inexpensive; HPLC: accurate but time‐consuming	Garza et al. 1999
Cocoa, roasted at 125°C–145°C (until uniform moisture)	Melanoidin and HMF concentrations, color	Spectrophotometer	Higher temperature, shorter time roasting: more melanoidins, less HMF; HMF formed after fluorescent precursors	Maillard reaction (advanced stage)	High‐MW melanoidins, HMF	Melanoidin: useful indicator of final stage NEB; HMF: formed via distinct pathways	Sacchetti et al., 2016
Dongbei Suancai, blanched at 100°C, fermented 30–40 days	Ascorbic acid, polyphenols, reducing sugar, amino N, HMF, absorbance at 410 nm	HPLC, spectrophotometer	Blanching delayed browning; fermentation enhanced browning due to polyphenol–sugar interaction	Ascorbic acid oxidation, polyphenol oxidation, Maillard	HMF, browning pigments	HPLC: precise, multi‐compound; Spectrophotometer: accessible but nonspecific	C. Wang et al. 2021
Crayfish tails, sterilized at 121°C, 7 min, stored at 37°C for 20 days	Color, Maillard products, free radicals, HMF	Colorimeter, UV–Vis spectrophotometer	Removal of hepatopancreas reduced lipid–Maillard interactions, lowering browning	Maillard reaction, lipid oxidation	HMF, Maillard pigments, radicals	UV–Vis: easy and fast; lacks molecular specificity	Q. Jiang et al. 2023
Spaghetti, dried at 60°C for 18–20 h	Color, furosine, HMF content	HunterLab colorimeter, HPLC	Furosine increased with lupin protein isolate, while HMF decreased	Early Maillard reaction	Furosine, HMF	Furosine: early NEB marker; HMF: later stage; Color: simple but subjective	Doxastakis et al. 2007
Krill oil, incubated at 20°C and 40°C for 28 or 42 days	Strecker products, pyrroles, free amino acids	GC–MS, spectrophotometer, LC–MS	Higher temperature enhanced lipid oxidation and accelerated NEB	Strecker degradation, lipid oxidation	Pyrroles, aldehydes	GC–MS/LC–MS: high sensitivity and specificity; costly and complex	F. S. H. Lu et al. 2014

### 2.2 Oxidation

Many of the reactions involved in the Maillard reaction involve the transfer of electrons, which is a characteristic feature of oxidation–reduction (redox) reactions. Oxygen is often involved in the Maillard reaction as a reactant or facilitator. Oxygen molecules can react with various reactive intermediates formed during the Maillard reaction, leading to the generation of additional flavor compounds and browning products (Shakoor et al. 2022; Starowicz and Zieliński 2019). For instance, the formation of reactive dicarbonyl compounds, such as 3‐deoxyglucosone, results from the oxidative degradation of Amadori rearrangement products. These dicarbonyls can further participate in oxidation‐driven transformations to produce furans, including 5‐HMF, through combined dehydration and oxidative cleavage of sugars. Strecker degradation is another key oxidative process, where amino acids react with dicarbonyls to yield Strecker aldehydes via oxidative decarboxylation. As the reaction progresses to the final stage, oxidative polymerization contributes to the formation of melanoidins.

For protein oxidation, excessive heat and prolonged cooking times can accelerate the oxidation of proteins, leading to the generation of off‐flavors and undesirable textures (Davies 2016; Domínguez et al. 2022; Stadtman and Levine 2000). Oxidative cleavage of proteins can result in the formation of undesirable bitter compounds from peptides (Weir 1992). Formation of protein carbonyls occurs when proteins react with reactive oxygen species (ROS), leading to the oxidation of amino acid side chains. Protein oxidation can lead to the formation of covalent cross‐links between amino acid residues. ROS (such as hydroxyl radicals and peroxides) can react with proteins to form protein hydroperoxides. These hydroperoxides can further contribute to oxidative damage and may serve as intermediates for other products (Gebicki 1997).

Two major protein oxidation products, carbonyls and Schiff bases, have been widely used as indicators of protein oxidation. The cooking process can increase the level of carbonyls, which can be used to indicate the level of protein oxidation. In one study that compared different cooking methods (boiling, steaming, microwaving, roasting, and frying) to prepare fish fillets, compared to the uncooked samples (2.1 nmol/mg protein), all the cooked samples increased in the number of carbonyl groups, with the highest increase of about fourfold when fried (9.7 nmol/mg protein) or roasted (10.6 nmol/mg protein) (Hu et al. 2017). Similarly, in another study that used different cooking methods of grilling, roasting, frying, and *Sous‐Vide* on jerky chicken, all the cooked samples had higher carbonyl concentrations compared to uncooked samples. The roasted samples had the highest carbonyl level (Silva et al. 2016). When pork was heat‐treated under the same temperature but for different lengths of time (10 and 30 min), longer heating time gave a greater rise in carbonyl levels (Traore et al. 2012).

Besides carbonyls, Schiff base formation is also a marker of protein oxidation during cooking. Schiff bases are generated during the reaction between protein side chain amino groups (from lysine, arginine, and histidine) and aldehydes (Chelh et al. 2007). Traore et al. showed that 30 min of cooking had a significantly higher concentration of Schiff bases compared with 10 min of cooking (Traore et al. 2012). Hu et al. found that among different cooking methods on fish fillets, roasting and frying showed the highest Schiff base level, followed by microwaving, boiling, and steaming (Hu et al. 2017). It was also investigated that when beef was cooked using superheated steam (400°C) for 300 s, the fluorescence emission from Schiff bases increased strongly; however, no significant impact was observed when using 67°C or 160°C steam (Gatellier et al. 2010). In general, protein oxidation caused by cooking processes and occurring during NEB is related to cooking methods, in which moist cooking processes such as boiling, steaming, and Sous‐Vide lead to lower protein oxidation, while dry cooking processes such as frying and roasting result in higher oxidation levels that vary depending on food matrix, temperature, and time.

### 2.3 Caramelization

**FIGURE 2:**
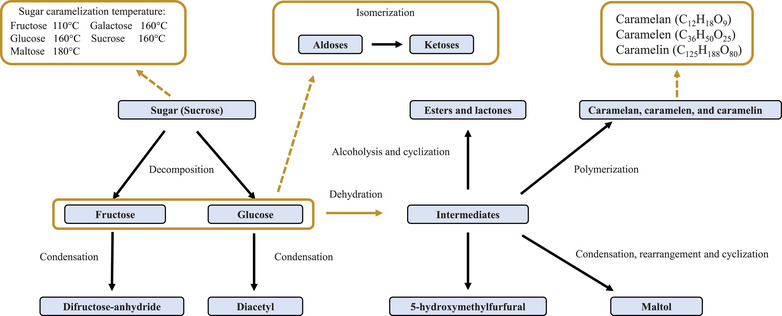
Summary of the caramelization process.

Caramelization is another common NEB reaction; it contains different reactions (Figure 2). Caramelization is a cooking process that leads to brown colors, burnt aromas, and flavors. Unlike the Maillard reaction, caramelization is pyrolytic and does not react with amino acids, and only carbohydrates are involved. It is widely employed in culinary practices to achieve a nutty, burnt flavor and a rich brown color in various dishes (Kroh 1994). In food systems where carbohydrates and proteins are jointly present, the Maillard reaction and caramelization may occur simultaneously.

Many reactions happen during caramelization, including hydrolysis, condensation, isomerization, dehydration, polymerization, and fragmentation (Kocadağlı and Gökmen 2016). Sucrose, for example, is first decomposed into fructose and glucose. These monosaccharides then undergo either condensation, to form difructose anhydride and diacetyl, or dehydration, to form different intermediates. These intermediate compounds have the potential to undergo a variety of chemical transformations, including alcoholysis, cyclization, polymerization, and condensation. These processes result in the formation of a diverse range of compounds such as esters, lactones, caramel polymers, HMF, and maltol. The brown colors produced during caramelization are associated with three groups of polymers, denoted caramelans, caramelens, and caramelins (Dalluge et al. 2014). These groups of caramel polymers are differentiated by their molecular weight (caramelan is the smallest, and caramelins are the largest).

Temperature, pH, and time play crucial roles in caramelization (Coghe et al. 2006). Higher temperatures accelerate caramelization, leading to faster browning and potentially deeper flavors. However, higher temperatures can also lead to the rapid development of excessive bitter compounds (Göncüoğlu Taş and Gökmen 2017). The caramelization temperature varies among the sugars: 110°C for fructose; 160°C for fructose, sucrose, galactose, and glucose; and 180°C for maltose. The pH can directly affect the stability of sugar molecules and their propensity to undergo caramelization. The speed of caramelization is typically at its minimum when the pH is approximately seven, but it accelerates in acidic (especially below pH 3) and basic (especially above pH 9) environments (Xu et al. 2023).

## 3 Effect of NEB on In Vitro Digestion of Starch and Protein

Due to the chemical changes that occur in starch and proteins during NEB reactions, the digestibility of these polymers can change. For example, the nutritional value of protein may be reduced during the early stages of the Maillard reaction, as amino acids react with reducing sugars, leading to a lower availability for absorption (ALjahdali and Carbonero 2019). Within caramelization, condensation reactions may cause decreases in starch digestibility. Several papers have studied the influence of heat treatment on starch digestibility and protein digestibility (some studies focused on pure proteins like β‐casein and β‐lactoglobulin). The examples of studies and their key findings are summarized in Table 2.

**TABLE 2:** Summarized findings for protein and starch digestibility in different nonenzymatic‐browning‐associated cooking methods.

Food material	Cooking conditions	Target nutrient	Method for digestibility	Main findings	Reference
Beef	Dry bath at 100°C for 0–45 min; oil bath at 270°C for 1 min	Myofibrillar proteins	Increase in soluble peptides	Longer cooking (5, 15, 30, and 45 min) reduced protein digestibility. Short cooking (100°C for 5 min or 270°C for 1 min) had minimal impact.	Santé‐Lhoutellier et al. 2008
Biscuits	Oven baking at 170°C and 120°C	Starch	Increase in reducing sugars	Higher temperature (170°C) baking led to higher starch digestibility than 120°C.	Conforti et al. 2012
Bread	Baked at 160°C–220°C for 9–20 min	Protein	Increase in free amino nitrogen	Higher baking temperature/time led to more crust browning, lower oxalate, and more minerals. Protein digestibility may increase but not linearly.	Bredariol et al. 2020
Bread	Baked at 150°C for 20 or 35 min	Protein	Increase in soluble peptides	Proofing increased digestibility, but baking decreased it. Higher digestibility linked to more nonfiber carbs; lower digestibility linked to more fiber/phytochemicals.	Wu et al. 2017
Milk	Heated at 90°C for 24 h (with/without glucose)	β‐Casein	SDS‐PAGE to reveal changes in molecular weight distribution	Heating without glucose increased digestibility; with glucose, digestibility decreased.	Pinto et al. 2014
Potato	Boiled (35 min), steamed (40 min at 103°C), microwaved (7 min at 750 W), grilled (45 min at 250°C)	Starch	Increase in glucose	Boiled potatoes had highest starch digestibility; grilled had lowest.	Narwojsz et al. 2020
Rice	Heated at 90°C for 0–48 h with glucose–glycine mixture	Starch	Increase in reducing sugars	Maillard reaction products reduced starch digestibility significantly.	Chung et al. 2012
Soybean	Heat‐treated at 100°C for 1–8 h	Protein	Residual insoluble nitrogen after digestion	Protein digestibility decreased with increasing oxidation from prolonged heating.	P. Lu et al. 2017

### 3.1 MRPs Can Alter Starch Digestibility by Inhibiting Digestive Enzymes

Various chemicals are produced during the Maillard reaction (Figure 1). The uniqueness and complexity of the Maillard reaction have attracted numerous studies, including those exploring its impact on starch digestibility. In a study focusing on the influence of MRPs on starch digestibility, the researchers subjected a mixture of glucose and glycine to heat at a temperature of 90°C for varying durations ranging from 0 to 48 h (Chung et al. 2012). This process yielded diverse levels of browning (measured by UV spectrophotometer), indicating different concentrations of MRPs. Then, they added various heated samples (containing MRPs) to the rice starch together with the amyloglucosidase to evaluate the effects of the MRPs on the starch hydrolysis rate. The findings revealed a substantial reduction in the efficiency of starch digestion upon the addition of MRPs from the Maillard reaction. Such reduction was attributed to the inhibition of α‐glucosidase activity by the MRPs through adding soluble starch to various concentrations of MRPs and measuring the degree of starch hydrolysis by the digestive enzymes. This corroborated an earlier investigation that indicated that the hydrolysis of starch was impeded due to the obstruction of α‐glucosidase‐active sites caused by thermally generated compounds, including MRPs (Schumacher et al. 1996). Similarly, another paper from this lab group showed the change in starch hydrolysis rates of gelatinized rice starch with the addition of MRPs (Chung et al. 2011). The inhibition effect was studied back in 1954, and the authors found that aldonolactones (produced during the NEB reactions) inhibited glycosidases and prevented carbohydrate breakdown due to their corresponding configuration (Conchie and Levvy 1957). In 1966, Kelemen and Whelan found that various polyols could inhibit the activity of glucosidases and galactosidases during NEB reactions by adding different concentrations of various polyols (Kelemen and Whelan 1966). Another study concluded that covalent modifications of protein‐bound amino acids and intermolecular and intramolecular cross‐linking can alter enzyme activity (Kroh and Schumacher 1996). A few papers have researched the mechanism of how NEB reaction products can inhibit amylolytic activity (Ademiluyi et al. 2019; Kroh and Schumacher 1996; Schumacher et al. 1996; Schumacher and Kroh 1994). Schumacher and Kroh found that Amadori compounds, such as fructosylglycine and maltulosylglycine, could affect the activity of certain enzymes, including α‐glucosidase and glucoamylase (Schumacher and Kroh 1994). Later, they found that such inhibition was present for the α‐glucosidase but not for the glucoamylase or α‐amylase, and this inhibition might be caused by the short‐chain α‐glucans (fructosylglycine and maltulosylglycine are Amadori compounds that can undergo further transformations leading to various reaction products, including short‐chain α‐glucans), which can bind to secondary binding sites and reduce hydrolysis (Schumacher et al. 1996). Note that α‐amylase is an endo‐amylase that breaks α‐(1,4)‐bonds within the interior of the starch chain, producing dextrins of varying lengths (van der Maarel et al. 2002). Glucoamylase and α‐glucosidase are amylases that target the external glucose residues of starch, but they differ in substrate preference: glucoamylase is more effective at hydrolyzing long‐chain polysaccharides, while α‐glucosidase favors short maltooligosaccharides (Struyf et al. 2017; van der Maarel et al. 2002). In the 21st century, Ademiluyi et al. used glucose–tyrosine MRPs, sucrose–tyrosine MRPs, fructose–tyrosine MRPs, and maltose–tyrosine MRPs to determine the antioxidant and enzyme inhibitory abilities (Ademiluyi et al. 2019). They confirmed the inhibitory effects of MRPs on α‐amylase and α‐glucosidase by comparing the enzyme activity of samples to a reference using absorbance‐based assays and expressing the results as percentage inhibition.

### 3.2 Impact of the Food Matrix on NEB and Protein Digestibility

Protein digestibility is not solely governed by the intrinsic structure of the protein but is strongly influenced by the surrounding food matrix, particularly under thermal processing, where NEB reactions (including the Maillard reaction and heat‐induced oxidation) are prevalent. Nutrients such as sugars and polyphenols, when present in protein‐rich foods, can participate in chemical reactions that modify protein structure, leading to aggregation, cross‐linking, or changes in enzyme accessibility. These interactions can impair proteolytic cleavage and limit amino acid release during digestion.

In model systems using isolated proteins, the presence of reducing sugars such as glucose can drastically alter the aggregation behavior of proteins during heat treatment through NEB reactions, especially via the Maillard reaction pathway. In one study, β‐lactoglobulin and β‐casein were incubated with glucose at 90°C for 24 h. The addition of glucose significantly modified the aggregation pathways of both proteins. For β‐casein, glucose facilitated the formation of large, spherical aggregates through covalent cross‐linking. Conversely, glucose appeared to inhibit fibril formation in β‐lactoglobulin, although aggregation still occurred through other mechanisms (Pinto et al. 2014). In both cases, protein–glucose interactions reduced enzymatic digestibility, likely due to steric hindrance and reduced accessibility of protease‐sensitive regions.

Beyond sugars, polyphenols (a group of plant‐derived antioxidants abundant in fruits, vegetables, and whole grains) also play a significant role in modulating protein digestibility. These compounds can undergo oxidation during NEB reactions, especially during heating, and form reactive quinones that interact with nucleophilic groups on proteins, such as thiol groups on cysteine residues. This results in covalent cross‐linking between protein molecules. For example, a study examined bread baked from selected gluten‐containing and gluten‐free flours (Wu et al. 2017). Pearson correlation analysis between bread composition and digestion rate indicated that, during peptide and amino acid evolution, protein–protein cross‐linking is the main chemical process reducing protein digestibility, and this cross‐linking is driven by total polyphenolic content. The presence of phenolic compounds was shown to promote cross‐linking of proteins because they can bind covalently to the sulfhydryl groups of the protein (R. Chen et al. 2011; Guo et al. 2021). In detail, phenolic compounds facilitate protein cross‐linking by undergoing oxidation to form highly reactive quinones, which covalently interact with sulfhydryl groups on cysteine residues in proteins, creating covalent bridges between different protein molecules and leading to structural modifications and aggregation (Guo 2021; Rawel and Rohn 2010). The reduction in free thiol content observed alongside protein cross‐linking suggests that protein polymerization or cross‐linking may be catalyzed by polyphenol‐mediated oxidation involving thiol residues (Lund 2021; Wu et al. 2017). The oxidation of polyphenols with an *o*‐diphenol structure to form *o*‐quinones causes them to behave as oxidants. In their oxidized form, *o*‐quinones are highly electrophilic and can readily react with nucleophilic groups, such as the thiol groups on cysteine residues in proteins. This oxidation of polyphenols, catalyzed by factors like oxygen or enzymes, makes them act as oxidants rather than antioxidants in these specific reactions, promoting cross‐linking or modifications of protein (Lund 2021). On the other hand, phenolic compounds possess antioxidant properties that can inhibit oxidation and the Maillard reaction. By scavenging free radicals, they reduce the extent of browning (Shakoor et al. 2022; Teng et al. 2018). In other words, polyphenols can exert both pro‐oxidant and antioxidant effects depending on the processing environment. Under thermal conditions, their oxidized forms may contribute to protein polymerization and reduced digestibility. However, under milder conditions, polyphenols may inhibit the Maillard reaction and oxidative damage by scavenging free radicals and stabilizing reactive intermediates.

Beyond glycation and polyphenol interactions, oxidative modifications act as an interconnected NEB pathway that further reduces protein digestibility. During thermal food processing, proteins are not only exposed to high temperatures but also to oxidative stress, which arises naturally during NEB reactions. NEB, particularly the Maillard reaction and caramelization, generates reactive intermediates and radicals that contribute to the oxidative modification of proteins. These oxidative changes can significantly impair protein digestibility by altering structure, reducing solubility, and blocking enzymatic access to cleavage sites. A key oxidative event during NEB is the oxidative deamination of lysine and arginine side chains, resulting in the formation of protein carbonyl groups. Lysine, in particular, is highly reactive not only in Maillard reactions (where it forms Amadori products) but also in oxidation, where it may form allysine and other aldehyde derivatives. These carbonylated residues reduce the number of available cleavage sites for proteases such as pepsin and trypsin, particularly in the case of endoproteases that target lysine or arginine residues (Domínguez et al. 2022). As a result, digestion efficiency is compromised.

Oxidative cross‐linking represents another significant pathway through which protein digestibility is reduced during NEB reactions. These cross‐links are formed under conditions of elevated temperature and oxidative stress, both of which are common during thermal food processing such as baking, roasting, or extrusion. In such environments, ROS and other oxidative agents are generated, often as byproducts of Maillard reactions or lipid oxidation (Decamps et al. 2016). These reactive species can modify specific amino acid side chains, particularly those of tyrosine and cysteine, triggering covalent bonding between protein molecules and leading to structural changes that hinder enzymatic access. One example of this process is the formation of dityrosine cross‐links, which occur when tyrosine residues are oxidized into tyrosyl radicals that subsequently couple together (Rodriguez‐Mateos et al. 2006; Aeschbach et al. 1976). This radical‐mediated reaction produces stable covalent bonds between tyrosine side chains, resulting in the formation of high‐molecular‐weight protein aggregates. These aggregates are notably resistant to proteolytic enzymes and often persist through both the gastric and intestinal phases of digestion, making them difficult to break down and absorb. Such dityrosine cross‐linking is enhanced under NEB conditions due to the combined effects of heat and oxidative intermediates. In parallel, thiol oxidation also contributes to oxidative cross‐linking by promoting the formation of disulfide bridges between cysteine residues. This process not only stabilizes the protein structure but also causes increased molecular rigidity and a reduction in conformational flexibility (Jiang, Carroll, et al. 2021; Fass and Thorpe 2018). As a result, digestive enzymes such as pepsin and trypsin encounter greater difficulty accessing and cleaving peptide bonds, especially in regions that require partial unfolding for enzymatic action.

In one study, channel catfish were treated with various cooking methods, including steaming and roasting. Cooking in general increased the in vitro protein digestibility compared to raw samples, where the digestibility was calculated based on the difference in protein content before and after digestion; however, among those cooking methods, steaming showed the highest digestibility, which was 66.78% after the intestinal phase. Roasting had a lower digestibility of around 62.7%. Additionally, roasted samples had the highest degree of oxidation, followed by steamed samples, based on carbonyl levels (Q. Jiang et al. 2022). This study also demonstrated that the degree of protein oxidation increased significantly as the food core temperature rose from 40°C to 90°C. This was measured by evaluating the levels of carbonyl groups and Schiff base, which are common intermediate products in NEB reactions. Lu et al. observed a continuous decline in protein digestibility as the level of oxidation increased during thermal processing. In their study, soybean meal was subjected to heat treatment at 100°C for varying durations ranging from 1 to 8 h. This heating condition facilitated the simultaneous progression of both Maillard reactions and oxidative modifications. The degree of protein oxidation was monitored through the measurement of protein carbonyl content, which served as an indicator of oxidative damage. As the carbonyl levels increased with longer heating times, a corresponding reduction in the digestibility of crude protein was recorded. Specifically, the in vitro protein digestibility decreased from 80.34% in the unheated control sample (0 h) to 74.00% after 8 h of heat treatment (P. Lu et al. 2017). Ji et al. investigated thermal‐induced interactions between soy protein isolate and malondialdehyde (MDA), where they found protein denaturation induced significant conformational changes that exposed reactive sites under thermal treatment (Ji et al. 2024). This structural unfolding facilitated increased interactions between MDA and the protein, intensifying oxidative modifications. As a result, protein digestibility decreased further due to increased oxidation and structural changes. This included a higher level of oxidized thiol groups and more extensive cross‐linking between protein subunits. Fluorescence analysis also showed stronger tryptophan quenching and greater fluorescence from MDA–protein adducts. These findings suggest that prolonged exposure to heat promotes oxidation‐associated structural changes in the protein matrix, such as cross‐linking and side‐chain modifications, which likely hinder enzymatic access during digestion (P. Lu et al. 2017). Therefore, protein oxidation during NEB is a critical determinant of digestibility. The interplay between oxidative stress, cross‐linking, and loss of reactive amino acids results in reduced enzymatic access, impaired hydrolysis, and lower nutritional quality of processed proteins (Hu et al. 2017).

While numerous in vitro and animal studies have demonstrated that Maillard reaction‐induced modifications to proteins impair digestibility by limiting enzymatic hydrolysis, it is important to distinguish between digestibility and systemic amino acid bioavailability. A recent human intervention study showed that dietary protein glycation can significantly compromise lysine bioavailability. Using intrinsically labeled milk proteins with low (3%) or high (50%) glycation levels, the authors reported a 63% reduction in the appearance of milk protein‐derived lysine in plasma after ingestion of the highly glycated protein (van Lieshout et al. 2025). Notably, despite the decrease in lysine bioavailability, whole‐body protein synthesis was still maintained in the short term due to the high protein dose (40 g) used in the study and the high lysine content of milk protein. However, under conditions where lysine or total protein is limited, the decrease in lysine bioavailability caused by the Maillard reaction may pose significant nutritional risks.

Taken together, these findings demonstrate that the Maillard reaction and oxidation are closely intertwined during thermal processing and are both influenced by food matrix components. Reducing sugars and polyphenols act not only as substrates but also as catalysts for structural modification via glycation and oxidation. The resulting changes, including carbonyl formation, thiol oxidation, dityrosine, and disulfide cross‐links, reduce the digestibility of proteins by limiting protease access and increasing structural rigidity. The extent of these reactions depends on processing conditions, such as temperature, duration, and moisture content, as well as the chemical nature and concentration of interacting nutrients.

## 4 Effect of NEB on In Vitro Fermentation

### 4.1 NEB Reactions Can Have Both Beneficial and Detrimental Effects on the Gut Microbiome

Several studies have provided evidence that individuals who regularly consume foods cooked using high‐heat cooking methods, such as roasting and grilling, exhibit a notable increase in the abundance of beneficial bacteria in their gut microbiota (Biddle et al. 2013; Ding et al. 2024; Lerma‐Aguilera et al. 2024; Pérez‐Burillo et al. 2018; W. Wang et al. 2023). The beneficial effects of NEB products on the gut microbiome are summarized in Table 3. Specifically, species belonging to *Ruminococcus* spp. and *Bifidobacterium* spp. were found in significantly higher quantities compared to individuals who predominantly consume foods cooked using gentler methods, such as boiling (Pérez‐Burillo et al. 2018).

**TABLE 3:** Summarized findings for beneficial effects of NEB products on the gut microbiome.

Food/chemical	Study focus	Method	Impacts on the gut microbiota	Reference
Fried and boiled meat (chicken, fish, and beef)	How different cooked meats impact the human fecal microbiota.	In vitro batch fermentation with human fecal microbiota	Higher numbers of *Clostridium* spp. in the microbiome from fried meat compared to boiled meats	Lerma‐Aguilera et al. 2024
Bread	Effects of overcooking on bread digestion and in vitro fermentation	In vitro batch fermentation with human fecal microbiota	Overcooking increased the abundance of several families with butyrate producers, including Ruminococcaceae and Oscillospiraceae. Overcooking decreased the relative abundances of *Sutterella, Parasutterella*, and *Escherichia*.	Ding et al. 2024
Beef patty	Effects of overcooking on beef patty digestion and in vitro fermentation	In vitro batch fermentation with human fecal microbiota	Overcooking decreased relative abundances of several genera from Pseudomonadota, including *Sutterella*, *Parasutterella*, unclassified Enterobacteriaceae, and unclassified Enterobacteroiales	Ding et al. 2024
Glycated protein (lactoferrin)	Influence of glycation on digestion and fermentation processes of lactoferrin in vitro	In vitro batch fermentation with human fecal microbiota	Glycated protein fermentation was associated with a higher relative abundance of Bacillota and a lower relative abundance of Pseudomonadota compared with non‐glycated protein fermentation	W. Wang et al. 2023
MRPs	The impact of MRPs on gut microbiota and metabolic profile during in vitro pig fecal fermentation	In vitro batch fermentation with pig fecal microbiota	MRPs derived from galactose and Galactooligosaccharides can reduce the growth of Pseudomonadota and *Bacteroides*	Liang et al. 2023
Melanoidins from bread	Melanoidins found in bread crusts hold promise as potential prebiotic components	In vitro batch fermentation with pig fecal microbiota	Bread crust melanoidins can work as potential prebiotic ingredients	Borrelli and Fogliano 2005
MRPs	The resilience of individual Maillard reaction products when exposed to the human colonic microbiota.	In vitro batch fermentation with human fecal microbiota	The human colonic microbiota has the capacity to degrade certain glycated amino acids, potentially employing them as sources of energy, carbon, and nitrogen	
CML	Effects of free and bound CML on gut microbiota and intestinal barrier in mice	In vivo 12‐week mouse study, feces collected	The study found that dietary‐free CML increased SCFA‐producing gut bacteria, while dietary‐bound CML increased *Akkermansia*. Dietary‐free CML may offer greater benefits for gut microbiota and SCFA production than dietary‐bound CML.	Yuan et al. 2024
MRPs	Maillard reaction products influence the composition of the gut microbiota of adolescents.	In vivo 2‐week human (adolescents) study, feces collected	Significant decreases in lactobacilli and Enterobacteriaceae following consumption of the MRP diet.	Seiquer et al. 2014

Our previous study compared the microbiome composition from the in vitro fermentation of overcooked beef and bread, displaying significant NEB in comparison with standard cooked samples (Ding et al. 2024). This study revealed that when the extra cooking time was applied, higher relative abundances of Ruminococcaceae were observed in the overcooked group compared with standard cooked, and higher relative abundances of Lachnospiraceae were found in the overcooked bread samples. Our study on overcooked bread found increased butyrate production during the fermentation of overcooked bread (Ding et al. 2024). High butyrate production could be linked to an increased relative abundance of butyrate‐producing Ruminococcaceae (Esquivel‐Elizondo et al. 2017). Butyrate has been reported to have several beneficial effects, including supporting normal intestinal function and regulating insulin secretion, inflammation, and lipid metabolism (den Besten et al. 2013; Puertollano et al. 2014). Liang et al. examined how MRPs sourced from galactose and galactooligosaccharides (added in hydrolyzed bighead carp meat) influence the microbial composition and metabolic profile of the intestine. Their results indicated that the MRPs derived from galactose and galactooligosaccharides may positively affect gut health by reducing the growth of Pseudomonadota (Liang et al. 2023). Later, Dou et al. examined the prebiotic potential of silver carp hydrolysate‐based MRPs using an in vitro fermentation model (Dou et al. 2025), which showed that moderate Maillard reaction increased short‐chain fatty acid (SCFA) production by 16.29% (*p *< 0.05) and significantly raised microbial diversity, along with a 264.24% increase in *Bacteroides*. Metabolomics identified elevated levels of indole‐3‐carboxaldehyde, indolepropionate, and glycochenodeoxycholic acid 3‐glucuronide, correlated with *Fusobacterium*, *Bacteroides*, and *unidentified_Clostridiales*. These results illustrate the dual effect of MRPs on gut microbiota—enhancing beneficial metabolites and taxa while also reflecting substrate‐ and condition‐dependent microbial shifts. One study found higher numbers of *Clostridium* spp. in the fermented slurry with fried meat compared to boiled meat after 5 and 48 h of fermentation (Shen et al. 2010). Another study concluded that glycated protein (lactoferrin) fermentation was associated with a higher relative abundance of Bacillota (formerly Firmicutes) and a lower relative abundance of Pseudomonadota (formerly Proteobacteria) compared with non‐glycated protein fermentation (W. Wang et al. 2023).

Several studies have investigated the effect of other MRPs on the gut microbiota (Borrelli and Fogliano 2005; Hellwig et al. 2015; Liang et al. 2023; Seiquer et al. 2014; Tuohy et al. 2006). A recent study investigated the impact of free and bound CML, formed during the NEB reaction between reducing sugars and the ε‐amino group of lysine, on gut microbiota and the intestinal barrier. This study found that free CML can increase the relative abundance of SCFA‐producing genera, including *Blautia*, *Faecalibacterium*, *Agathobacter*, and *Roseburia* (Yuan et al. 2024), while the dietary‐bound CML can increase the relative abundance of *Akkermansia*, which has anti‐inflammatory properties and is potentially beneficial to intestinal health (Zhai et al. 2019). Another study examined the stability of dietary MRPs when exposed to the intestinal microbiota (Hellwig et al. 2015). This study incubated human fecal samples with four different MRPs, including *N*‐ε‐fructosyllysine (FL), CML, pyrraline (PYR), and maltosine (MAL), for 72 h. After 4 h of fermentation, FL could not be detected anymore. After 24 h of fermentation, around 40% CML and 20% PYR were degraded. No significant degradation of MAL was found during fermentation. This study first demonstrated that the human colonic microbiota has the capability to break down specific glycated amino acids, potentially utilizing them as a source of energy, carbon, and nitrogen. Filipp et al. later confirmed that probiotic bacteria, primarily lactic acid bacteria, can metabolize the Amadori rearrangement product, FL, by monitoring their ability to degrade FL. Quantitative experiments revealed that FL was fully broken down into lysine (Filipp et al. 2024). Furthermore, using ^13^C_6_‐labeled FL as a substrate, it was shown that the sugar moiety of the Amadori product is broken down into lactic acid, marking the first evidence that certain lactic acid bacteria can use the sugar moiety as a substrate for lactic acid fermentation (Filipp et al. 2024).

Another study showed that bread crust melanoidins can work as potential prebiotic ingredients by increasing the abundance of *Bifidobacterium* (Borrelli and Fogliano 2005). In this study, different formulas and sizes of bread were cooked at 220°C for 20 or 60 min. Pronase E in Tris‐HCl was added to the freeze‐dried bread crust to release the high‐molecular‐weight melanoidins, which were then collected through ultrafiltration after centrifugation. Extracted melanoidins, fecal slurry, and selective media were used to do the anaerobic fermentation for 24–120 h. In general, they found that anaerobic and facultative anaerobic bacteria, including *Lactobacillus* spp., *Bifidobacterium*, Enterobacteriaceae, *Bacteroides* spp., and Clostridia, can utilize bread melanoidins as carbon and nitrogen sources, with *Bifidobacterium* showing notable growth. The growth of these microorganisms varies across different melanoidin‐containing samples, highlighting that the initial ingredients and processing conditions significantly influence the prebiotic properties of bread melanoidins.

A human study reported that MRPs can modulate gut microbiota composition in adolescents (Seiquer et al. 2014). In this study, 20 male adolescents participated in a 2‐week randomized crossover trial, where they were provided with two distinct diets: a white diet, emphasizing foods cooked without the Maillard reaction or typically free of MRPs, and a brown diet, featuring processed foods with noticeable browning and rich in MRPs. Each diet was consumed for 1 week, followed by a 40‐day washout period, and then another week of consumption. After each dietary phase, fecal samples were collected from each participant for subsequent analysis. The fecal samples showed significant decreases in lactobacilli (a generic term for bacilli that are lactic acid bacteria) and Enterobacteriaceae following consumption of the brown diet. Lactobacilli are generally considered beneficial bacteria in the gut, as they can help maintain a healthy balance by producing lactic acid and supporting digestion. However, Enterobacteriaceae were considered the harmful microbiome associated with inflammatory bowel diseases (IBDs) (Baldelli et al. 2021).

Similarly, other studies have also found some negative effects of NEB reaction products on the gut microbiota, which are summarized in Table 4. For example, glycated bovine serum albumin (BSA) promoted a potentially harmful community structure, with increases in sulfate‐reducing bacteria and decreases in *Bifidobacterium* compared with native BSA (Mills et al. 2008). In another study that also used BSA as a substrate, fecal samples from both healthy people and ulcerative colitis (UC) patients were used for in vitro fermentation with substrates including heated BSA, glycated BSA, and native BSA. Compared to native BSA, glycated BSA caused an increase in *Clostridium perfringens* and sulfate‐reducing bacteria, while decreasing *Bifidobacterium* and *Lactobacillus*, using microbiotas collected from both healthy individuals and UC patients (Tuohy et al. 2006). When NEB intake was restricted, the relative abundance of *Prevotella copri* significantly decreased in their stool samples (Yacoub et al. 2017). In other words, NEB (which contains AGEs) may help maintain the relative abundance of this organism, which has been linked to an increased susceptibility to arthritis (Scher et al. 2013). However, some studies have suggested that *Prevotella* is generally considered beneficial in modern diets, as it may help inhibit the development of type 2 diabetes (Sharma et al. 2022; Tsai et al. 2023).

**TABLE 4:** Summarized findings for the harmful effects of NEB products on the gut microbiome.

Food/chemical	Study focus	Method	Impacts on the gut microbiota	Reference
Heated BSA and glycated BSA	The effects of dietary MRP on gut microbiome	In vitro batch fermentation with human fecal microbiota	Heated BSA had higher numbers of the detrimental bacteria and lower numbers of bifidobacteria and lactobacilli compared to native BSA	Tuohy et al. 2006
Glycated BSA	The effect of native, heated, and glycated BSA on the UC and non‐UC colonic microbiome	In vitro three‐stage continuous flow bioreactor with human fecal microbiota	Glycated BSA regulated the microbiota of UC patients detrimentally by increasing harmful bacteria such as Clostridia, *Bacteroides*, and SRB, as well as decreasing beneficial ones including eubacteria and bifidobacteria	Mills et al. 2008
Chickpeas, bread, pepper, banana, and chicken cooked in different methods	The effect of different cooking methods on gut microbiome	In vitro batch fermentation with human fecal microbiota	Intense cooking methods increased the abundance of beneficial bacteria (such as *Bifidobacterium* spp. and *Ruminococcus* spp.) compared to mild cooking methods; for some foods, intense cooking decreased the healthy bacteria	Pérez‐Burillo et al. 2018
AIN‐93G diet added with 3% glucose–lysine	Whether defined Maillard reaction compounds are the reason for the effect of MRP	In vivo 87‐day mouse study	Dietary MRPs can alter the intestinal microbiota composition of rats (decrease in lactobacilli and total bacteria counts); specific effects are probably due to the amount of intake and the structure of MRP	Seiquer et al. 2014
AIN‐93G diet with 10% bread crust	The impact of diets with MRPs from bread crust on gut microbiome and short‐chain fatty acids (SCFAs)	In vivo 87‐day mouse study	Dietary MRPs are fermented by gut microbiome in vivo, which can change bacterial composition and SCFA production; in mice fed with diets with bread crust, there was a decrease in log_10_ counts of *Lactobacillus* spp. and *Bifidobacterium* spp. and an increase in *Escherichia*/*Shigella* counts	Delgado‐Andrade et al. 2017
CML	The effect of oral ingestion of CML on two experimental IBD models and mice gut microbiota	In vivo 21‐day mouse study	Oral intake of CML did not cause IBD and had small influence on gut microbiota (increase in Bacteroidaceae and decrease in Lachnospiraceae)	ALJahdali et al. 2017
Regular chow added with methylglyoxal–BSA or heated regular chow	Association between gut microbiota and glycometabolic profiles in mice fed with high‐AGE diets	In vivo 24‐week mouse study	High‐AGE diet–fed mice showed a decrease in butyrate producers including Bacteroidales family S24‐7, Ruminococcaceae, and Lachnospiraceae	J. Wang et al. 2022
Heated AIN‐93G diet	Impact of AGEs on cecal microbiota and colon permeability	In vivo 6‐, 12‐, and 18‐week mouse study	AGE treatment impacted the gut microbiome in a harmful way by reducing the relative abundance of Bacteroidetes, together with declining *Alloprevotella* and Ruminococcaceae at the genus level; colon permeability might partially increase	Qu et al. 2017
Heated AIN‐93G diet	Impact of dietary AGEs on mice gut microbiota	In vivo 8‐month mouse study	High AGE ingestion reduced the α‐diversity of microbiota and changed its composition by increasing *Helicobacter* level	Qu et al. 2018
Low‐AGE diet	The effects of dietary AGEs on the gut microbiome of end‐stage renal disease patients	In vivo 1‐month human study	The restriction of dietary AGE intake changed the gut microbiome, with a decrease in the relative abundance of *Prevotella copri* and *Bifidobacterium animali*s, as well as an increase in *Alistipes indistinctus*, *Clostridium citroniae*, *Clostridium hathewayi*, and *Ruminococcus gauvreauii*	Yacoub et al. 2017

In the study by Pérez‐Burillo et al., which examined different foods and cooking methods for in vitro fermentation, it was found that toasted bread with more Maillard reactions showed a lower relative abundance of *Roseburia*, *Coprococcus*, *Blautia*, *Butyricimonas*, *Anaerostipes*, *Clostridium* cluster XIVa, *Clostridium* cluster XIVb, and *Ruminococcus* at the genus level compared to un‐toasted bread. In contrast, the abundance of *Collinsella* and *Parasutterella* increased, with the latter being associated with Crohn's disease and other potentially harmful health effects. The paper also found that compared to raw bananas, which contained few MRPs, fried and roasted bananas resulted in a lower abundance of *Bifidobacterium*, *Barnesiella*, and *Butyricimonas*, while increasing *Parasutterella*, *Oscillibacter*, *Roseburia*, and *Coprococcus*. Among the increased bacteria, *Parasutterella* is considered harmful, and *Oscillibacter* is another harmful bacterium associated with an increased risk of obesity (Pérez‐Burillo et al. 2018), while *Roseburia* and *Coprococcus* are considered beneficial bacteria because of their ability to generate butyrate (Rivière et al. 2016). This study illustrates a mix of both beneficial and harmful effects, offering insights into the dual nature of the observed outcomes.

There are also several other studies that used mouse models to study the effects of MRPs on gut microbiota. Seiquer et al. provided mice with an AIN‐93G diet supplemented with 3% glucose–lysine. The microbiome from the cecal samples indicated a significant decrease in lactobacilli (generic term referring to bacilli) in high‐MRP diet–fed mice compared to control mice (Seiquer et al. 2014). In another study, bread crust, a major source of MRPs, was added at 10% to an AIN‐93G diet (a standardized rodent diet developed by the American Institute of Nutrition in 1993, specifically formulated for the growth of laboratory rodents like rats and mice) and fed to rats for 87 days. The results indicated that the consumption of a diet with bread crust significantly decreased *Lactobacillus* and *Bifidobacterium* counts in the cecal samples of the rats, compared to control AIN‐93G‐fed mice (Delgado‐Andrade et al. 2017). Additionally, when healthy mice orally ingested CML for 21 days, a significant decrease in Lachnospiraceae and *Sutterella* was found in fecal samples compared to mice that received saline (ALJahdali et al. 2017). Lachnospiraceae was associated with many beneficial health effects, including butyrate production (Zhang et al. 2019), while *Sutterella* was linked to autism spectrum disorder (L. Wang et al. 2013).

There are also several studies that used AGEs as part of the diet for mouse models, learning about the impact on gut microbiota. Wang et al. fed mice exogenous AGEs by adding methylglyoxal–BSA to their chow or by heating the regular chow to create dietary AGEs. Compared to the regular chow‐fed group, it was found that the heated chow group had significantly decreased the diversity of the gut microbiota. The relative abundance of some butyrate‐producing bacteria families, such as Ruminococcaceae and Lachnospiraceae, decreased in the feces of mice that were fed the high‐AGE diet (J. Wang et al. 2022).

Another mouse study used a heated diet that had high AGEs as the feeding for mice. Compared to the regular (low AGEs) diet, the relative abundance of Bacteroidetes, *Alloprevotella*, and Ruminococcaceae declined in the cecal samples from the high‐AGE‐diet group. The authors also found a significant drop in acetate and propionate from cecal content in the high‐AGE‐diet group (Qu et al. 2017). The authors conducted similar research using the same high‐AGE diet for mice but with a longer study duration (8 months compared to 18 weeks). They found a significant reduction in the abundance of *Rikenellaceae*, Lachnospiraceae, and *Desulfovibrionaceae* at the family level and a decrease in *Desulfovibrio*, *Rikenellaceae* RC‐9 group, unclassified Lachnospiraceae, *Alistipes*, and Lachnospiraceae at the genus level. In terms of SCFAs, both acetate and butyrate decreased following AGEs intake, which slightly differs from the findings in their previous study. This difference may be attributed to the longer feeding period (8 months) in this study compared to the 18 weeks in their prior research (Qu et al. 2018).

Different MRPs as fermentation substrates can inhibit or promote distinct bacterial groups. While some studies report drawbacks of MRPs on the gut microbiome and others note beneficial shifts, available evidence remains limited. Only a few works use in vitro digestion fermentation models to assess their effects on gut health. Reported negative outcomes are often mixed, with some microbes benefiting and others suppressed. This complexity highlights the context‐dependent nature of MRPs, shaped by interactions with food compounds and individual microbiota. Since MRPs vary across foods, and other compounds can also alter microbial balance, contradictory findings remain unresolved. Further research should clarify how specific MRPs and co‐occurring components influence microbial metabolism and determine conditions under which MRPs are harmful or beneficial.

### 4.2 Proteins Become Less Fermentable due to Cross‐Linking

Protein cross‐linking, particularly as a result of thermal processing and chemical reactions such as Maillard browning or oxidation, significantly alters the digestibility and fermentability of dietary proteins. While many studies have emphasized that cross‐linking impairs enzymatic digestion in the upper gastrointestinal tract, its downstream effects on microbial fermentation in the colon are equally important. Cross‐linked proteins are more resistant to proteolytic cleavage, which can lead to two outcomes: increased delivery of undigested protein to the colon and decreased microbial access to those proteins due to their altered structure.

Under typical conditions, when proteins escape digestion in the small intestine, they enter the colon and become substrates for microbial fermentation. However, protein fermentation in the human gut is considered harmful to the human body because it can produce chemicals that are detrimental (Windey et al. 2012). First, it can produce ammonia from the deamination of amino acids, and high levels of ammonia can be toxic and may contribute to liver and kidney damage (Russell et al. 1991). Hydrogen sulfide is another harmful compound produced from the fermentation of sulfur‐containing amino acids like cysteine and methionine (Windey et al. 2012). Hydrogen sulfide is a toxic gas that can damage the colonic mucosa and has been implicated in the pathogenesis of IBD (Stummer et al. 2023). Additionally, phenols and indoles are produced from the fermentation of certain amino acids like tyrosine and tryptophan (Diether and Willing 2019; Smith and Macfarlane 1997; Windey et al. 2012). They are associated with an unpleasant odor and have been linked to an increased risk of colorectal cancer (Hughes et al. 2000; Liu et al. 2023). Consequently, reducing the availability of proteins for fermentation in the colon may be beneficial in lowering the generation of such toxic byproducts.

Interestingly, protein cross‐linking can influence this dynamic in two contrasting ways. On the one hand, poorly digested proteins due to cross‐linking could increase the protein load reaching the colon. On the other hand, the same cross‐linking that impairs enzymatic digestion in the small intestine may also make proteins less accessible to colonic microbes. This reduced fermentability may limit the proliferation of proteolytic bacteria and decrease the generation of harmful fermentation products. This may help explain the contradictory results observed regarding their effects on the human gut microbiome. A study focused on the influence of overcooking on in vitro fermentation concluded that the relative abundance of Pseudomonadota, a phylum of many proteolytic bacteria, was significantly decreased in the overcooked whole wheat bread and beef samples (Ding et al. 2024). Meanwhile, this study found fewer free amines in overcooked samples than in standard cooked samples. In addition to their involvement in the Maillard reaction, free amines are a reactive site for protein cross‐linking. This process of protein cross‐linking can diminish the availability of proteins for fermentation and ultimately lead to a decrease in the relative abundance of proteolytic members of the gut microbiota, including bacteria from the Pseudomonadota phylum. Likewise, in another investigation, the fermentation of lactoferrin glycated with chitooligosaccharides (oligosaccharides of chitosan) was linked to a decreased relative abundance of Pseudomonadota in comparison to the fermentation of non‐glycated proteins. Simultaneously, there was an increased relative abundance of Bacillota (Ding et al. 2024; W. Wang et al. 2023).

Taken together, these findings suggest that while protein fermentation generally raises health concerns, NEB‐induced cross‐linking may reduce the fermentability of proteins, thereby limiting the formation of harmful byproducts and altering gut microbial composition. This may partially explain the inconsistent observations regarding the effects of heated or browned foods on the gut microbiome. Cross‐linking reduces protein availability for fermentation, thereby decreasing the selective advantage of proteolytic bacteria and potentially mitigating the negative consequences of protein fermentation.

## 5 Health Issues

Although this review has discussed several of the potentially beneficial effects of MRPs on the gut microbiota, there have been some studies showing negative effects of MRPs on human health independent of the gut microbiota. There are three major types of oxidation products that may pose health risks. The first group is AGEs, formed by reactions between reactive carbonyls (from sugars or oxidized starch/lipids) and amino groups in proteins. During food processing, particularly under high‐temperature conditions, AGEs accumulate and can subsequently be ingested as dietary AGEs. While endogenously formed AGEs are strongly implicated in chronic diseases (Byun et al. 2017; Uribarri et al. 2007), the health effects of dietary AGEs remain less clear and depend on intake, absorption, and metabolism. Approximately 10% of dietary AGEs are absorbed in healthy individuals, and about 30% of this absorbed fraction is excreted in urine (Nowotny et al. 2018). Intervention studies suggest that although tissue AGE levels may rise with high‐AGE diets, acute exposure does not necessarily lead to measurable health effects (Nogueira Silva Lima et al. 2024). Furthermore, evidence from animal models indicates that diet‐induced AGE accumulation in tissues may be reversible (van Dongen et al. 2021). A 4‐week intervention in individuals with obesity showed that a diet high in AGEs had no significant effects on glucose metabolism, lipid profiles, blood pressure, or systemic inflammation (Linkens et al. 2022).

Another major type of oxidation products from NEB that can cause health issues is ROS. MRPs have been shown to induce oxidative stress by stimulating intracellular ROS production or impairing antioxidant defense mechanisms. This oxidative stress can result in widespread cellular damage, including lipid peroxidation, protein oxidation, and DNA strand breaks, ultimately leading to inflammation and the deterioration of cellular integrity (Hanukoglu 2006). ROS generated in the context of NEB may alter the function of key metabolic enzymes by modifying their active sites or disrupting cofactor interactions. Such enzyme dysfunction can interfere with crucial physiological pathways, including those involved in energy production, detoxification, immune responses, and cellular signaling. Over time, these disruptions may contribute to metabolic imbalance and promote the development of chronic diseases (Alfadda and Sallam 2012; Brieger et al. 2012; Q. Chen et al. 2018; Datta et al. 1999).

Carcinogenic compounds produced during oxidation reactions are another major group of substances that raise significant health concerns. Some studies have suggested a potential link between MRPs and an increased risk of cancer (Farhadian et al. 2012; Mottram et al. 2002; Virk‐Baker et al. 2014). When food is cooked at high temperatures or processed extensively, MRPs with carcinogenic properties may form. These compounds, such as acrylamide (Pelucchi et al. 2006) and polycyclic aromatic hydrocarbons (Mastrangelo et al. 1997), have been associated with an elevated risk of certain types of cancer, including gastrointestinal, lung, and breast cancer. Prolonged exposure to MRPs through the consumption of charred or overcooked foods may contribute to this risk. Consuming foods rich in MRPs, particularly those that are heavily processed or charred, may also lead to digestive system discomfort (Pedreschi and Murkovic 2017). These adverse effects from charred food can significantly impact the quality of life of those with pre‐existing digestive conditions, such as irritable bowel syndrome (IBS) or IBD. These people are more likely to experience GI discomfort from eating charred food compared with people who do not have IBS or IBD (Teresa Pacheco et al. 2018).

Finally, MRPs may reduce the bioavailability of essential nutrients. Glycation can interfere with amino acid availability by modifying lysine residues, as discussed previously (van Lieshout et al. 2025). In addition to amino acids, the formation of MRPs during NEB can reduce the availability of certain vitamins, such as thiamin (Kathuria et al. 2023).

Collectively, moderate intake of MRP from cooked foods appears to be safe, although thermal processing can reduce the content of certain nutrients through degradation. While in vitro and cellular studies demonstrate the capacity of MRPs to induce oxidative stress, their detrimental effects are not necessarily observed in human studies.

## 6 Conclusion

This review explores the NEB reactions, their influence on protein and starch digestibility and fermentation, and the effects of NEB reactions on the gut microbiome, highlighting both beneficial and detrimental impacts. While NEB reactions have been associated with some benefits for gut microbiota, such as reduced protein fermentation, these findings must be balanced with evidence suggesting potential risks to human health.

However, there are still gaps in understanding how those different NEB reactions influence the bioavailability of starch and protein, and which reactions predominantly influence the health of the human gastrointestinal tract. Meanwhile, a systematic cross‐sectional comparison of different cooking methods is highly necessary to understand how they can affect the digestion and fermentation of proteins and starch.

## Nomenclature

AGEs: advanced glycation products
BSA: bovine serum albumin
CML: *N*‐ε‐carboxymethyllysine
FL: *N*‐ε‐fructosyllysine
GC–MS: gas chromatography–mass spectrometry
HMF: 5‐hydroxymethylfurfural
HPLC: high‐performance liquid chromatography
IBD: inflammatory bowel disease
IBS: irritable bowel syndrome
LC–MS: liquid chromatography–mass spectrometry
MAL: maltosine
MDA: malondialdehyde
MRPs: Maillard reaction products
NEB: nonenzymatic browning
PYR: pyrraline
ROS: reactive oxygen species
SCFA: short‐chain fatty acid
UC: ulcerative colitis

## Author Contributions

**Wensheng Ding**: conceptualization, investigation, visualization, writing–original draft. **Yichen Bai**: investigation, writing–review and editing. **Devin J. Rose**: conceptualization, investigation, writing–review and editing, project administration, resources, supervision.

## Conflicts of Interest

The authors declare no conflicts of interest.
